# Spatial ability contributes to memory for delayed intentions

**DOI:** 10.1186/s41235-020-00229-2

**Published:** 2020-08-08

**Authors:** Veit Kubik, Fabio Del Missier, Timo Mäntylä

**Affiliations:** 1 Department of Psychology, Bielefeld University, D-33615 Bielefeld, Germany; 2 Department of Psychology, Stockholm University, Stockholm, Sweden; 3 Department of Life Sciences, University of Trieste, Trieste, Italy

**Keywords:** Prospective memory, Multitasking, Spatiotemporal hypothesis, Spatial ability, Executive functioning

## Abstract

Most everyday activities involve delayed intentions referring to different event structures and timelines. Yet, past research has mostly considered prospective memory (PM) as a dual-task phenomenon in which the primary task to fulfill PM intentions is realized within an ongoing secondary task. We hypothesized that these simplified simulations of PM may have obscured the role of spatial relational processing that is functional to represent and meet the increased temporal demands in more complex PM scenarios involving multiple timelines. To test this spatiotemporal hypothesis, participants monitored four digital clocks, with PM deadlines referring either to the same clock (single-context condition) or different clocks (multiple-context condition), along with separate tests of spatial ability (mental rotation task) and executive functioning (working memory updating). We found that performance in the mental rotation task incrementally explained PM performance in the multiple-context, but not in the single-context, condition, even after controlling for individual differences in working memory updating and ongoing task performance. These findings suggest that delayed intentions occurring in multiple ongoing task contexts reflect independent contributions of working memory updating and mental rotation and that spatial relational processing may specifically be involved in higher cognitive functions, such as complex PM in multiple contexts or multitasking.

## Significance

A central issue for our modern society is how we can deal with the higher demands of concurrent information processing that comes with recent developments in digital technology (i.e., smartphones, social media). Specifically, the ability to simultaneously form, monitor, and remember multiple delayed intentions within limited time frames plays a critical role in our everyday lives (e.g., scheduling a meeting before lunch, making a ticket reservation, and remembering to buy milk on the way home). While previous research mainly considered prospective memory (PM) as a dual-task phenomenon, involving the task to (repeatedly) fulfill a single intention in the context of another ongoing task (cf. taking medication every third hour), this is the first study to examine a more complex form of PM in the dynamic context of ongoing multiple tasks. On the basis of a new time-based PM paradigm, results showed that spatial ability (as measured by mental rotation performance) uniquely contributed to PM for intentions embedded in multiple (compared to a single task) context. We suggest that pure reliance on simplified dual-task paradigms of PM may have obscured the role of other nonexecutive functions. Because remembering multiple intentions, specifically across different event structures, increases temporal complexity and task coordination demands, spatial-relational processing may be a computationally effective mechanism for handling higher demands in executive functioning. One novel and important theoretical implication of this study is that spatial ability is also engaged in PM of intentions in multiple task contexts, supporting the more general role of spatial ability in nonspatial and time-related cognition.

## Spatial ability contributes to memory for delayed intentions

With recent developments of the digital society, demands involving the scheduling and interleaving of multiple activities have increased significantly for both children and adults (Rideout, Foehr, & Roberts, 2010). The amount of time people spend on engaging in multiple tasks has increased dramatically during the last decades. We have to plan and coordinate multiple goals and intentions in different task contexts and time frames, often delayed and interrupted by other everyday activities. Yet, virtually no prior studies on more complex PM have investigated how we form, monitor, and remember delayed intentions in a single task context versus multiple ongoing task contexts.

Research on PM has been very active and innovative during the last decades, especially since the development of laboratory paradigms, which have been instrumental for conceptual advancement (for overviews, see Brandimonte, Einstein, & McDaniel, 2014; Kliegel, Mackinlay, & Jäger, 2008; McDaniel & Einstein, 2007). In this influential prior work, PM has been considered as a dual-task phenomenon, partly mirroring more traditional research on attention and (retrospective) memory. Indeed, most past studies of PM have investigated how we form and remember a *single* PM intention (primary task) within a *single* ongoing task context (secondary task), such as remembering to press the space bar once every minute in the context of a lexical decision task or identifying people wearing glasses in the context of a face recognition task. Note that these PM intentions often need to be repeatedly remembered (cf. taking medication every third hour). However, the task is one-dimensional in the sense that PM intentions refer to one and the same event structure or timeline of the ongoing task (here referred to as *single*-*context PM*). In contrast, everyday activities often involve multiple ongoing task contexts or “cognitive threads” (Salvucci & Taatgen, 2008); that is, delayed intentions can be embedded within different task domains (referred to as *multiple context PM*). Thus, past research on PM might lack generality due to its reliance on overly simplified simulations of everyday activities.

Prominent theories suggest distinct processes to support PM that are typically clustered under the terms of strategic monitoring and spontaneous retrieval. The framework of Preparatory Attentional and Memory Processes (Smith, 2003, 2016) assumes that PM is mainly driven by controlled executive functioning processes in terms of strategic monitoring. The multiprocess framework (Anderson, McDaniel, & Einstein, 2017; Scullin et al., 2018; Shelton & Scullin, 2017), however, assumes that PM performance is additionally guided by automatic, nonstrategic processes and may rather reflect a dynamic interplay of both processes. A general implication of these frameworks is that individuals with reduced executive functions—for example, due to cognitive aging (Schnitzspahn, Stahl, Zeintl, Kaller, & Kliegel, 2013) or frontal lobe damage (Burgess, Gonen-Yaacovi, & Volle, 2011)—have greater difficulties in more complex PM tasks than individuals with more efficient control functions. The aim of this study was not to contrast theories but rather to extend these influential views of PM; we focus on task conditions of PM in which executive control functions might be complemented by other cognitive skills—in the case of multiple-context conditions, by spatial abilities.

In line with prior research, we assume that executive functioning is the main source of individual and developmental differences in PM (Mackinlay, Kliegel, & Mäntylä, 2009; Mäntylä, Carelli, & Forman, 2007). However, this reliance on simplified simulations of PM may have obscured other sources of individual differences in PM. Indeed, most experimental simulations of PM involve dual-task performance in that PM deadlines are embedded along with a single event structure or timeline. However, to better reflect more complexities of PM in everyday life, these experimental simulations should also examine delayed intentions embedded in multiple ongoing tasks. In a similar vein, it should be noted that a majority of the research referred to as “multitasking” is also based on dual-task paradigms (for an overview, see Koch, Poljac, Müller, & Kiesel, 2018), focusing on cognitive bottlenecks (e.g., Pashler, 1994; Schubert, 1999), crosstalk between input and output mechanisms (e.g., Janczyk, Renas, & Durst, 2017), and task order control of two temporally overlapping tasks (e.g., Kübler, Reimer, Strobach, & Schubert, 2018). However, everyday multitasking differs from typical dual-task paradigms, in terms of both number of component tasks and overall duration of the multitasking scenario (see also Burgess, Veitch, de Lacy Costello, & Shallice, 2000; Logie, Trawley, & Law, 2011; Mäntylä, 2013; Redick et al., 2016). Compared with monitoring and scheduling multiple ongoing activities, demands for temporal monitoring are very low in most dual-task conditions, including single-context PM, in which delayed intentions are typically embedded within the temporal structure of the same ongoing task context.

Given the assumption that temporal complexity is a characteristic feature of time-based PM (and multitasking), remembering delayed intentions across independent task contexts is expected to require a higher degree of monitoring and coordination of deadlines along separate timelines and cognitive threads. Following this line of reasoning, remembering future intentions may involve higher cognitive processes other than executive functioning. Indeed, prior research showed that we understand and handle rather basic aspects of time (e.g., duration, sequence) by translating them in a spatial reference frame (Bender & Beller, 2014; Bonato, Zorzi, & Umiltà, 2012; Boroditsky, 2000; Casasanto & Boroditsky, 2008; Dehaene & Brannon, 2011; Núñez & Cooperrider, 2013). On the basis of this notion, we propose a *spatiotemporal hypothesis* of more complex forms of PM. We suggest that representing complex temporal patterns of deadlines as spatial relations is a basic and computationally efficient cognitive strategy because it alleviates cognitive control demands and possibly provides complementary processing advantages. That is, individuals with more effective spatial abilities can better meet the high temporal demands when handling immediate and delayed intentions in multiple contexts by relying on these additional spatial-relational processes. Consistent with earlier work in different areas of cognitive sciences, support for this “time in space” relational process has been observed in the field of multitasking. Several studies provided evidence that spatial ability, as measured by the mental rotation task (MRT), and executive functioning, as measured by the critical component of working memory (WM) updating, make independent contributions to multitasking performance (Mäntylä, 2013; Mäntylä, Coni, Kubik, Todorov, & Del Missier, 2017; Todorov, Del Missier, Konke, & Mäntylä, 2015; Todorov, Del Missier, & Mäntylä, 2014; Todorov, Kubik, Carelli, Del Missier, & Mäntylä, 2018).

Because these studies involved monitoring and coordination of multiple tasks with minimal demands on both prospective and retrospective memory, we aimed here to test the primary predictions of the spatiotemporal hypothesis within a complex PM paradigm by manipulating the complexity of the PM context. A methodological challenge in investigating PM in multiple contexts is that PM intentions should refer to different (vs. same) subtasks while eliminating differences in other task-related factors, including task complexity and expertise. Furthermore, for a fair contrast between PM involving a single task versus multiple task contexts, the ongoing task should be the same in both conditions. Also, the subtasks should be virtually identical and independent in that monitoring one task should not provide information about the state of the other tasks. As far as we know, no earlier studies have contrasted complex PM performance in single versus multiple contexts while minimizing task-specific differences and eliminating cross-task alignment.

In the present individual-differences study, we used a time-based PM paradigm in which delayed intentions were embedded in an ongoing task. The latter involved multiple simultaneous tasks rather than a single ongoing task as in most laboratory simulations of PM. We increased the complexity of the ongoing task for three reasons. First, as noted earlier, many everyday activities involve multiple ongoing activities with a mix of actions and intentions, rather than an isolated task (such as making lexical decisions), in which delayed intentions are embedded. Second, because PM performance is typically related to ongoing task costs (McDaniel & Einstein, 2007; Smith, 2003), the background activities should be virtually identical between the single-context and multiple-context conditions to allow a fair comparison. Third, we intended to examine PM performance of varying context complexity (PM intentions related to a single context vs. multiple contexts) in a setting of high cognitive load in which attentional resources for task management and temporal processing are shared with the ongoing task. If participants need to temporally monitor, update, and switch among different ongoing tasks, executive control and temporal processing are already taxed and should interfere more with scheduling and tracking of multiple intentions in complex PM performance (cf. McNerney & West, 2007; Occhionero, Esposito, Cicogna, & Nigro, 2010). Thus, potential differences in recruiting these limited cognitive resources should become more accentuated when participants attempt to additionally meet the increased temporal demands of PM tasks in a multiple (compared to single) task context. Finally, this task arrangement provided an opportunity to test the predictions of the spatiotemporal hypothesis in the context of multitasking performance (possibly replicating our earlier work) and of delayed intentions (possibly extending the hypothesis to more complex PM).

In this study, participants monitored four digital clocks, or more specifically counters, and needed to respond whenever one of the differently colored counters displayed readings that fulfilled a specific rule (e.g., multiples of 20; see also the Methods section for details). To prevent cross-task monitoring, the clocks ran at different rates (see also Mäntylä, 2013). Embedded in these ongoing monitoring activities, participants were instructed to remember and execute PM intentions (i.e., to press the space button) at certain deadlines (represented by the specific readings of the counters). In the single-context condition, participants needed to remember the PM intentions referring to the deadlines within one and the same counter; for example, they needed to respond when the counter showed “110,” “210,” and “310.” In the multiple-context condition (manipulated within subjects), participants needed respond whenever any of three different counters reached any of the deadlines. That is, participants had to respond when, for example, the red, blue, or yellow counters would show the time reading of “104.” Specifically, participants needed first to monitor the (occluded) counters, then to activate the counter with an approaching deadline, and finally to respond in time when the target reading appeared in the counter. We used four different sets of PM deadlines, and their temporal distribution was the same in both conditions. Participants also completed separate tasks of spatial ability (as measured by MRT) and WM updating (as measured by the matrix-monitoring task).

Following the spatiotemporal hypothesis and our earlier work, we expected that individual differences in both the matrix-monitoring performance and MRT performance would predict PM performance and that these contributions would be selective. More specifically, we expected that individual differences in matrix-monitoring performance would predict PM performance and, more importantly, that MRT performance would incrementally explain variability only in PM performance in the multiple-context condition in which remembering delayed intentions becomes more complex in terms of temporal monitoring and coordination. However, we expected that individual differences in MRT performance would not, or at least would to a lesser degree, contribute to single-context PM performance. We assumed that transforming within-context temporal relations (i.e., before vs. after along the same timeline) to spatial relations would not bring additional computational benefits for remembering PM intentions.

## Methods

Data, codebook, and analysis scripts are available at the following public repository: https://osf.io/3njp8/.

### Participants

Power analysis for a multiple regression with maximum of four predictors was conducted in G*Power to determine a sufficient sample size using an alpha of 0.05, a power of 0.80, and a medium effect size (*f*^2^ = 0.15; Faul, Erdfelder, Buchner, & Lang, 2009). Based on these criteria, the desired sample size is 85. One hundred twelve Stockholm University undergraduates participated in exchange for movie vouchers or course credit. They were between 19 and 49 years of age, with an equal number of men (mean [*M*] = 27.107 years, standard deviation [*SD*] = 6.017) and women (*M* = 28.232 years, *SD* = 7.066).

### Materials

*PM* was assessed with a time-based paradigm, consisting of an ongoing monitoring task and a PM task. In the *ongoing task*, participants monitored four upward-running number counters (1, 2, 3, … *∞*), representing digital clocks. In this *counter task* (Mäntylä, 2013), participants needed to press the spacebar whenever one of the counters showed a digital reading that fulfilled a simple rule (e.g., when the last two digits of counter 1 were a multiple of 25 or the last two digits were identical). Because the counters were occluded by colored fields, participants needed first to press a color-coded key. This enabled them to monitor the reading of the counter for 2 s and then eventually to press the corresponding response key when the deadline appeared. To prevent the four component tasks from being handled as a unitary task, the counters ran at different rates (i.e., 1.9 s, 3.7 s, 5.72 s, or 2.3 s per unit; for more details, see Todorov et al., 2015).

In the *PM task*, participants needed to remember the PM intention (i.e., pressing the spacebar) at three different points in time (i.e., deadlines). Because the counters were designed to simulate differently paced digital clocks, participants needed to remember the PM intention and to meet the deadlines along one temporal task context versus three independent temporal task contexts. In the single-context condition, the three deadlines referred to the same (ongoing) task context (e.g., the green counter) and were to be remembered at specific time readings (e.g., “110,” “210,” or “310”). In the multiple-context condition, the three deadline referred to three different (ongoing) task contexts (e.g., the red, blue, and yellow counters) with the same time reading across all task contexts (e.g., “104”). Specifically, participants needed to monitor the counter first and then to press the target button at the specific point in time when the PM intention was to be remembered and executed. The distance of the PM deadlines to the next ongoing task targets was more than 9 s within the same counter and more than 2 s between counters. Proportion of correct responses was the dependent measure of PM performance.

*Spatial ability* was assessed with the Vandenberg and Kuse MRT (Peters et al., 1995; Vandenberg & Kuse, 1978). After receiving written instructions and completing four practice items, participants were given 3 min to complete the 12 items of set A. Each participant was given one 12-item MRT (A) subset of the paper-and-pencil test for 3 min.^1^ Each of the 12 items comprised five three-dimensional cube constructions that were displayed in various orientations. With the left-most figure as the target figure, the task was to identify the two (out of four) figures to the right-hand side that matched the target figure; the remaining two figures were distractors. Participants were supposed to mentally rotate the figures and to mark the two matching figures to the right. Responses were scored as correct only if both stimulus figures matching the target figure were identified. The proportion correct out of 12 items was the dependent measure.

*Working-memory updating* was assessed with the matrix-monitoring task (Salthouse, Atkinson, & Berish, 2003). It is assumed to be one key component of executive functioning (Miyake et al., 2000) and considered to be specifically relevant for time monitoring (e.g., Mäntylä et al., 2007). In each of 16 trials, two 4 *×* 4 matrices were displayed for 2.5 s followed by a 500-ms interstimulus interval (ISI) on the computer screen—one above and one below a black line. A black dot was located in each of the two matrices, followed by a series of four arrows both below and above the line for 1.2 s, followed by a 250-ms ISI. Both dot location and arrow direction were independent for the two matrices, and their presentation order was randomized for each participant. The arrows—pointing either up, down, left, or right—indicated for the participants to “move” the dot in their head in the distance of one cell in the respective direction. Thus, upper (lower) arrows indicated the direction of the imaginary movement of the upper (lower) matrix. At the end of each trial, only one of the matrices was probed, with equal probability across trials. Presented with a dot in one cell of the matrix, participants needed to decide whether the dot was moved in the correct position (by pressing the “z” key) or the wrong position (by pressing the “m” key). Participants practiced the matrix-monitoring task with an easier version that presented only a single matrix on each trial. Proportion of correct responses was the dependent variable.

### Design

Context complexity was manipulated in a within-subjects design with a single-task context and a multiple-context condition. To alleviate potential item effects, we created two equivalent sets of deadlines (i.e., time readings) for the two context conditions at which the PM intention was to be executed; we assigned set 1 to group 1 (*n* = 56; 28 men and 28 women), and set 2 to group 2 (*n* = 56; 28 men and 28 women). To reduce counter-specific effects, there were two counterbalanced versions of each set of deadlines. In the single-context condition, the PM intention was to be executed at three different deadlines in either counter 1 or counter 4. In the multiple-context condition, this PM intention was to be executed at three different deadlines, but referring to the same time reading at the three different counters that were not used in the single-context condition. For each group, we assigned context condition and time reading set to the two task sessions in a counterbalanced order, separately for women and men (but no effects of or interaction with set version were observed; *p*s > .10). Importantly, the three PM deadlines across context conditions and sets were equivalent with respect to their temporal placement and the temporal distance to the reading events of the ongoing task (i.e., the PM deadlines and ongoing task events were always separated for at least 2 s, and their relative temporal distance was the same across tasks). Proportion of correct responses was the dependent measure of PM performance.

### Procedure

Informed consent was obtained before participation, and the study was completed according to the ethical guidelines established by the Declaration of Helsinki. Each individually tested participant completed a 2-h session. Participants completed the first PM task, followed by the MRT and the second PM task, and finally the matrix-monitoring task. As is typical for an individual-difference approach (cf. Wilhelm, Hildebrandt, & Oberauer, 2013), the overall task order was the same for all participants. Except for the MRT, all the tasks were computerized, and the stimuli were presented on a 20-inch display. Each task included separate instructions and a practice phase, during which the experimenter checked that participants had properly understood the instructions. Participants first had 1-min study sessions to repeatedly learn the deadlines when the PM intention had to be executed (i.e., the three readings of the counter) and to subsequently recall them within 2 min, until perfect recall was achieved. After completing a 1-min practice trial of the ongoing task, and after the experimenter confirmed that participants had understood the instructions, they were again asked to recall the previously learned PM deadlines. If necessary, participants relearned the PM time points for perfect recall. Then, participants were instructed on the PM task that followed. The latter was 10 min long, during which the PM intention was to be executed thrice after a delay period of more than 2½ minutes, followed by a retrospective memory test in which participants had to immediately recall and write down the three PM deadlines within 2 min. Following the matrix-monitoring task, participants studied the second set of critical PM deadlines for 1 min until correct recall was achieved. After a brief period of instructions, the second PM task followed. The two PM tasks were virtually the same, except that the PM time deadlines belonged either to the same or to different (ongoing) task contexts, respectively. At the end, participants immediately responded to a background questionnaire.

## Results

Because prior research did not show any systematic effects (or trade-offs) between monitoring frequency and response accuracy (Mäntylä, 2013; Todorov et al., 2015; Todorov et al., 2018), we used the latter measure as the primary dependent variable. PM responses were considered correct if they were within one digit of the PM deadlines (e.g., counter readings “109,” “110,” and “111” would be considered correct responses if the PM deadline was “110”). Ninety-two participants showed correct (retrospective) recall of all six PM deadlines, and the remaining 18 participants failed in recall in either the single-context or multiple-context condition (or in both; *n* = 2). Retrospective recall of PM deadlines was somewhat lower in the single-context condition (*M* = 2.829, *SD* = .502) than in the multiple-context condition (*M* = 2.928, *SD* = .293), but this difference was not significant, *F*(1, 110) = 3.339, *p* = .070, ω^2^ = .021.

The following analyses were based on two-tailed tests and on the whole sample, except that one participant was excluded because of very low performance in both PM (.00) and retrospective recall of PM time points (with five of six failures). We also completed separate analyses after excluding participants with recall failures in PM time points (*n* = 19), but these analyses showed a pattern of results similar to the findings reported here. On the basis of our primary hypothesis, we examined the relation between MRT and PM performance with correlation and regression analyses.

Table 1 summarizes the correlation data for the main measures. These correlations show that both matrix-monitoring and MRT performance were associated with PM performance, but that the strength of their associations varied with the context condition. Specifically, matrix-monitoring performance showed nonsignificant correlations with PM scores in the single-context condition (*r* = .182, *p* = .056) and multiple-context condition (*r* = .161, *p* = .091) that did not differ significantly (*Z* = − 0.175, *p* = .861). However, MRT performance showed a significant correlation with PM performance in the multiple-context condition (*r* = .428, *p* < .001) but not in the single-context condition (*r* = .156, *p* = .103); the correlations differed significantly in size as a function of context complexity (*Z* = 2.421, *p* = .015). It can also be noted that the ongoing task data per se showed associations similar to the ones observed in the PM data in the multiple-context condition, with significant correlations with both matrix-monitoring performance (*r* = .278, *p* = .003) and MRT performance (*r* = .355, *p* < .001).

**Table 1:**
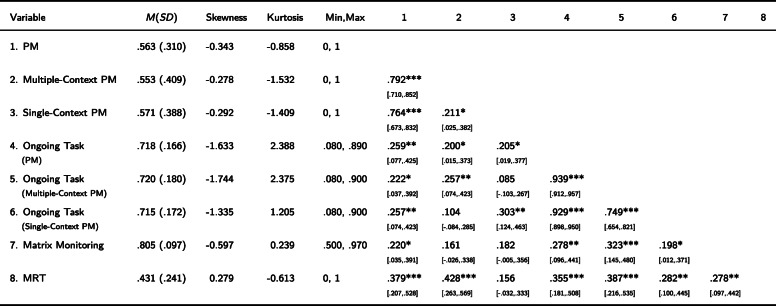
Descriptive statistics of all measures and their correlations (*N* = 111)

*Note*. *p < .05, **p < .01, ***p < .001 (2-tailed); MRT = Mental-Rotation Task; PM = Prospective Memory

### Prospective memory

To further assess the contribution of these individual differences measures to variability in PM performance, we conducted separate hierarchical regression analyses for the average PM data as well as for the PM data in the single-context and multiple-context conditions. In these analyses, matrix-monitoring performance was entered first, followed by MRT performance in the second step and ongoing task performance in the third step (to control for potential trade-offs). As a final step, we entered single-context PM performance in the hierarchical regression analyses on multiple-context PM performance to examine whether the more basic abilities still account for reliable amounts of variance when controlling for single-context PM aspects. The results of this series of hierarchical regression analyses are displayed in Table 2.

**Table 2:**
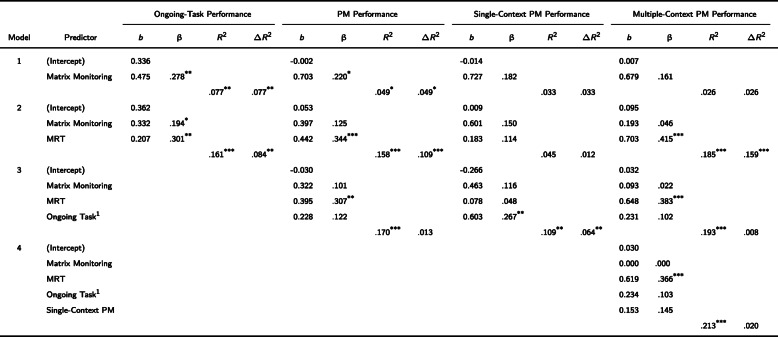
Hierarchical regression of the main measures on both PM Performance and Ongoing-Task Performance

*Note*. *p < .05, **p < .01, ***p < .001 (2-tailed); MRT = Mental-Rotation Task; PM = Prospective Memory; ^1^Ongoing-Task = Ongoing-task performance corresponding to PM performance, in the multi-context condition, single-context condition, and across both conditions (PM performance), respectively

Results showed that matrix-monitoring performance contributed to average *PM performance* (Model 1). Critically, MRT performance remained the only significant predictor after accounting for matrix-monitoring performance (Model 2) and also for ongoing task performance (Model 3). Similar results were revealed for *multiple-context PM performance*, except that the contribution of matrix-monitoring performance did not reach significance (β = .161, *p* = .091; Model 1). More importantly, MRT performance still predicted unique variance and was a significant predictor even when single-context PM performance was entered as a final predictor (Model 4). For *single*-*context PM performance*, results showed that neither matrix-monitoring performance made a significant contribution (β = .182, *p* = .056; Model 1), nor did MRT significantly contribute (β = .114, *p* = .247; Model 2). Ongoing task performance was the only predictor that explained incremental variance of single-task PM performance (β = .267, *p* = .006; Model 3).

### Ongoing task performance

Hierarchical regression analyses were conducted on ongoing multitask performance by entering matrix-monitoring performance (in the first step) and MRT performance (in the second step) as predictors. Consistent with prior research, the results showed that matrix-monitoring performance was a reliable predictor of ongoing task performance (Model 1) and that MRT performance made an incremental contribution (explaining an additional 8.361% of the PM variance; see Model 2).

### General discussion

The starting point of this study was the observation that most past studies have simulated everyday PM by relying on a single, repeated PM intention within a single-task context. We hypothesized that these laboratory simulations might have obscured other sources of individual differences in PM. We hypothesized that remembering delayed intentions across multiple ongoing tasks (or cognitive threads) requires a higher degree of scheduling and temporal tracking than within the same ongoing task. This difference in temporal complexity might increase the contribution of spatial-relational processes, which are not typically observed in single-context conditions. This should be particularly the case if the PM task is embedded in an ongoing multitask context with a high temporal processing demand.

The findings of this study supported this line of reasoning in that participants with good spatial abilities showed better performance in multiple-context PM than those with less effective spatial abilities. By contrast, individual differences in MRT were not significantly related to PM performance in a single-context condition. Furthermore, regression analyses showed that MRT incrementally predicted PM performance in the multiple-context condition, but not in the single-context condition, even after controlling for the contributions of matrix-monitoring and ongoing task performance. These results suggest that spatial-relational processes contributed to performance in more complex forms of PM in the service of meeting the increased temporal processing demands. However, the single- and multiple-context conditions showed similar overall levels of both PM and ongoing-task performance. These findings suggest that although the multiple-context condition was temporally more complex than the single-task condition, the involvement of WM updating was similar and nonsignificant in both conditions. Note, however, that there was a significant relationship between WM updating and average PM (likely due to increased power), in line with prior research, suggesting that executive functioning correlates with PM in more complex scenarios (Martin, Kliegel, & McDaniel, 2003) or in nonfocal conditions of event-based PM (Brewer, Knight, Marsh, & Unsworth, 2010). Critically, participants recruited additional spatial-relational processes in the multiple-context condition only. These selective effects may suggest that the high executive control demands involved in complex goal-directed tasks, such as multiple-context PM and multitasking, can be alleviated in terms of a spatial offloading mechanism (Todorov et al., 2018; for an overview on cognitive offloading, see Risko & Gilbert, 2016). However, more research is needed to gain a deeper understanding of this theoretical important issue.

The present study also revealed that spatial ability (mental rotation performance) explained incremental multitasking performance above and beyond WM updating, replicating the results pattern of previous studies (Kubik, Zimmermann, Del Missier, & Mäntylä, 2020; Mäntylä, 2013; Mäntylä et al., 2017; Todorov et al., 2018). In addition, we have shown that WM updating was related to the performance of the ongoing multitask. This corroborates prior research showing spatial WM to be a reliable predictor of multitasking, both using the counter paradigm (Mäntylä, 2013; Mäntylä et al., 2017; Todorov et al., 2015, 2018) and also in diverse multitasking paradigms (SynWin, control tower, air-traffic-control lab, Redick et al., 2016; Edinburgh Virtual Errands Test, Logie et al., 2011). Taken together, this pattern of results replicates and extends prior studies on multitasking, in which (prospective and retrospective) memory demands varied. It provides support for the spatiotemporal hypothesis in complex goal-directed tasks, such as PM for delayed intentions and multitasking.

Although our results suggest that spatial ability contributes to multiple-context PM (and ongoing-multitasking) performance beyond the WM updating component of executive control functions, it should be noted that the spatiotemporal hypothesis of multitasking is constrained by conceptual boundary conditions. A central assumption of the hypothesis is that individual differences in MRT contribute to task performance when the demands on temporal coordination are relatively high. These demands, in turn, are related to individual characteristics and task conditions. In most everyday situations of task coordination (cf. preparing a breakfast, Rendell & Craik, 2000), overlearned scripts, schemas, and related knowledge structures reduce demands on planning and task coordination by providing a spatiotemporal structure for guiding goal-directed actions. Furthermore, even when the support of these knowledge structures is reduced (as in our counter task), demands on temporal coordination can be low, for example, due to few component tasks (as in dual-tasking) or because the temporal constraints are rather flexible or marginal (as in reactive tasks, in which participants respond to unpredictable PM cues). In these conditions, demands or possibilities for temporal monitoring and coordination are very limited, and therefore individual differences in MRT have no, or at least have less, room to contribute to PM performance.

The present findings support the hypothesis that spatial-relational processing contributes to temporal coordination of multiple immediate (ongoing task performance) and delayed intentions (PM performance), possibly through some form of “time-in-space” relational processes. In comparison, PM performance in simpler, single-task contexts and also other kinds of temporally less demanding “multitasking” scenarios requiring coordination of only component tasks were not associated with individual differences in MRT (cf. Strayer, Medeiros-Ward, & Watson, 2013). This results pattern supports the notion that the increasing temporal coordination demands from two to more cognitive threads may be an important hypothetical boundary condition of the spatiotemporal hypothesis (cf. Mäntylä & Todorov, 2013). To test this notion further, in more recent studies, we manipulated the number of tasks in a multitasking paradigm by simultaneously presenting either two or four counters at a time (Kubik et al., 2020). Both dual-tasking and multitasking were correlated significantly with MRT, though the latter exhibiting a stronger association. To measure differences in temporal coordination demands, multitasking costs (i.e., difference scores between dual-tasking and multitasking performance) were calculated. Critically, mental rotation performance was the main predictor of multitasking costs and was explained variability in multitasking above and beyond WM measures (Kubik et al., 2020).

Taken together, these results provide a first hint that a significant spatial involvement is observed after a certain degree of task/context complexity. This complexity level was reached in PM only when being embedded in multiple contexts but not in a single context (representing a dual-task phenomenon); however, it was already reached in temporally more demanding “multitasking” scenarios when handling at least two component tasks simultaneously. Thus, taken together with the evidence from more recent studies, there seems to be no general hypothetical boundary condition of the spatiotemporal hypothesis from two to more cognitive threads on a task level. Instead, the involvement of spatial-relational processes may rather reflect the quantitative amount in temporal coordination demands; for example, PM in multiple-task contexts (relative to a single-task context) and multitasking (relative to dual-tasking) may involve only *more* spatial-relational processing. However, considering the current state of knowledge, this notion remains speculative at this point, and additional research is needed to gain a deeper understanding. For the type of spatial processes involved, there is some evidence supporting the notion that in addition to mental rotation performance, coordinate (rather than categorial) processing (cf. Jager & Postma, 2003; cf. Newcombe & Huttenlocher, 2000) can incrementally explain multitasking above and beyond executive functioning (Todorov et al., 2015) and/or WM capacity (Kubik et al., 2020). Future research should elucidate with a latent-variable approach whether spatial ability per se or only specific spatial abilities contribute to individual differences in multiple-context PM (and complex multitasking per se), considering, for example, spatial perception and spatial visualization (cf. Carroll, 1993; Linn & Petersen, 1985) or dynamic versus static spatial abilities (cf., Newcombe & Shipley, 2015; Uttal et al., 2013).

The present work is purely correlational and does not warrant any causal claims. Future work should aim to demonstrate the direct (translational) effect of spatial processes in complex multi-context PM performance and its (potentially alleviating) effect of executive control via experimental studies. Furthermore, the exact nature of these postulated spatial-relational processes needs to be further elucidated; they may involve specific spatial-relational processes and/or rather more abstract transformational mechanisms (e.g., Walsh, 2003). Future PM research should also focus on these complex and more ecological forms of PM, both by more systematically varying the number of ongoing task contexts and by manipulating the number of PM intentions, which has been scarcely examined. In one of the rare studies, Craik and Bialystok (2006) reported a simulation study in which participants had to remember to start and stop cooking five foods so that all the foods would be ready at the same time. This task involved multiple intentions in terms of different deadlines, but it should be noted that the timers referring to the five foods were simultaneously visible and running at the same rates. Thus, participants could actually handle these multiple deadlines as a dual-PM task by aligning the individual cooking times along the same timeline. Future research should more systematically investigate the PM task by manipulating the number of PM intentions and/or the ongoing task contexts; this would better simulate the critical dimensions of everyday task conditions and lead to a better understanding of the underlying strategic processes of PM. To conclude, our study supports the central role of spatial ability in higher-order cognition, including complex PM and multitasking, possibly resonating its predictive validity in the fields of science, technology, engineering, and mathematics (Newcombe, 2016).

## Data Availability

Materials, quantitative data, codebook, and analysis scripts are made available on the open repository osf.io.

## Abbreviations

ISI: Interstimulus interval
M: Mean
MRT: Mental rotation task
PM: Prospective memory
SD: Standard deviation
WM: Working memory

## Footnotes

1 The MRT (D) subset was administered for half of the participants but was then omitted because of repeated reports of high difficulty. These data were not included in the final analyses, owing to floor effects.
